# The Association of Periodontitis and Peripheral Arterial Occlusive Disease in a Prospective Population-Based Cross-Sectional Cohort Study

**DOI:** 10.3390/jcm10102048

**Published:** 2021-05-11

**Authors:** Nicole Jacobi, Carolin Walther, Katrin Borof, Guido Heydecke, Udo Seedorf, Ragna Lamprecht, Thomas Beikler, Sebastian E. Debus, Christoph Waldeyer, Stefan Blankenberg, Renate B. Schnabel, Ghazal Aarabi, Christian-Alexander Behrendt

**Affiliations:** 1 Department of Prosthetic Dentistry, Center for Dental and Oral Medicine, University Medical Center Hamburg-Eppendorf, 20246 Hamburg, Germany; n.jacobi@uke.de (N.J.); c.walther@uke.de (C.W.); k.borof@uke.de (K.B.); g.heydecke@uke.de (G.H.); u.seedorf@uke.de (U.S.); r.lamprecht@uke.de (R.L.); g.aarabi@uke.de (G.A.); 2 Department of Periodontics, Preventive and Restorative Dentistry, University Medical Center Hamburg-Eppendorf, 20246 Hamburg, Germany; t.beikler@uke.de; 3 Epidemiological Study Center, University Medical Center Hamburg-Eppendorf (UKE), 20246 Hamburg, Germany; 4 Department of Vascular Medicine, University Heart and Vascular Center UKE Hamburg, Research Group GermanVasc, University Medical Center Hamburg-Eppendorf, 20246 Hamburg, Germany; s.debus@uke.de; 5 Department of Cardiology, University Heart and Vascular Center UKE Hamburg, University Medical Center Hamburg-Eppendorf, 20246 Hamburg, Germany; c.waldeyer@uke.de (C.W.); s.blankenberg@uke.de (S.B.); r.schnabel@uke.de (R.B.S.); 6 German Centre for Cardiovascular Research (DZHK), Partner Site Hamburg/Kiel/Lübeck, 20246 Hamburg, Germany

**Keywords:** peripheral arterial disease, periodontitis, cohort studies, population health, health services research, smoking, cardiovascular disease, risk factors, ankle brachial index, diabetes

## Abstract

Objectives: Peripheral arterial occlusive disease (PAOD) and periodontitis are common chronic diseases, which together affect almost 1 billion people worldwide. There is growing evidence suggesting a relationship between chronic inflammatory conditions such as periodontitis and PAOD. This study aims to determine an association between both entities using high quality research data and multiple phenotypes derived from an epidemiological cohort study. Design: This population-based cross-sectional cohort study included data from 3271 participants aged between 45 and 74 years enrolled in the Hamburg City Health Study (NCT03934957). Material & Methods: An ankle-brachial-index below 0.9, color-coded ultrasound of the lower extremity arteries, and survey data was used to identify participants with either asymptomatic or symptomatic PAOD. Periodontitis data was collected at six sites per tooth and included the probing depth, gingival recession, clinical attachment loss, and bleeding on probing index. Multivariate analyses using logistic regression models were adjusted for variables including age, sex, smoking, education, diabetes, and hypertension. Results: The baseline characteristics differed widely between participants neither affected by periodontitis nor PAOD vs. the group where both PAOD and severe periodontitis were identified. A higher rate of males, higher age, lower education level, smoking, diabetes, and cardiovascular disease was observed in the group affected by both diseases. After adjusting, presence of severe periodontitis (odds ratio 1.265; 97.5% CI 1.006–1.591; *p* = 0.045) was independently associated with PAOD. Conclusion: In this cross-sectional analysis of a prospective cohort study, an independent association between periodontitis and PAOD was revealed. The results of the current study emphasize a potential for preventive medicine in an extremely sensitive target population. Future studies should determine the underlying factors modifying the relationship between both diseases.

## 1. Introduction

Peripheral arterial occlusive disease (PAOD) is a common manifestation of systemic atherosclerosis. In addition to its clinical aspects that limit the patient’s quality of life, it has a profound cost impact on the healthcare system [1]. In 2015, more than 230 million patients were affected worldwide with an increasing prevalence of approximately 15–20% in patients aged between 70 and 74 years and further increasing prevalence with age [2,3]. Furthermore, millions of diabetics may be included in this target population [1]. In addition to the fact that approximately 25% of patients with symptomatic PAOD have a concomitant diabetes [1], it is commonly known that diabetics frequently develop peripheral vascular complications and chronic limb-threatening ischemia in the longer term. Even higher numbers were reported for chronic inflammatory diseases of the oral cavity. In 2010, periodontitis was the sixth most common disease worldwide and affected 743 million people [4]. The global age-standardized prevalence of the severe course of periodontitis reached 11% between 1990 and 2010 [4]. Both diseases have remarkable overlaps concerning risk factors such as higher age, smoking, hypertension, and a history of coronary artery disease and stroke [2,5,6,7]. A major shared risk factor for both periodontitis and PAOD [8] is the growing burden of diabetes. The International Diabetes Federation estimated that 700 million people of the global population will be living with diabetes until 2045 [9]. Diabetes increased the risk of developing periodontitis by 86% [10] and individuals with both diabetes and periodontitis have higher systemic inflammation when compared with healthy controls [11]. The underlying pathogenic pathways are certainly bidirectional. Advanced glycation of end-products in diabetes patients activate the local immune system and increase cytokine levels, which consequently lead to local tissue degradation, thereby boosting the clinical phenotype of periodontitis [12]. Vice versa, subgingival bacteria can enter vascular lesions and contribute to the overall systemic inflammation, which may induce insulin resistance [12]. In addition to diabetes-related vascular complications leading to chronic limb-threatening ischemia and limb loss, the independent role of diabetes as concomitant disease has been discussed recently [13]. Thus, there is growing evidence for a multifactorial inflammatory genesis, again emphasizing the relation between both diseases [14]. However, only a few studies have so far determined the relationship between periodontitis and PAOD [15,16,17,18].

The aim of the current study was to determine if periodontitis is independently associated with PAOD using well-characterized phenotypes in a cross-sectional epidemiological cohort study in Germany.

## 2. Material & Methods

### 2.1. Subjects, Study Design and Setting

The Hamburg City Health Study (HCHS) is a large, prospective, long-term, population-based cohort study and a unique research platform to obtain substantial knowledge about several important risk and prognostic factors in major chronic diseases. Participants between 45 and 74 years of age from the general population of Hamburg, Germany, are taking part in an extensive baseline assessment at one dedicated study center [19].

The ethics committee of the Medical Association of Hamburg approved the study protocol (PV5131) and the study was registered at ClinicalTrial.gov (NCT03934957). This manuscript was prepared according to the STROBE guidelines [20].

### 2.2. Assessment of Dental Variables

Dental examination, performed by certified study nurses, included the following steps: periodontitis was diagnosed with a standardized periodontal probe (Hu-friedy Mfg. Co., LLC, Chicago, USA) following a full mouth—six sites protocol, excluding the third molars. Decisive periodontal parameters are the probing depths, the bleeding on probing index (BOP), the oral plaque index (PI), and the gingival recession. The respective clinical attachment loss (CAL) per tooth was calculated. The grading of periodontitis in severity grades was based on the classification of Eke & Page [21]. Subsequently the DMFT (D = decayed, M = missing, F = filled, T = teeth) was calculated. Participants requiring endocarditis prophylaxis prior dental examination were excluded.

### 2.3. Assessment of Peripheral Arterial Occlusive Disease

The dichotomized variable PAOD was derived from structured anamnesis data, self-based questionnaire, and baseline examination. All participants were asked if they had experienced any history of intermittent claudication, ischemic rest pain, or ischemic wound healing disorders. At the study center, the ankle-brachial-index (ABI) was measured in both legs and cut off for diagnosis were values below 0.9 [22]. Furthermore, a peripheral color-coded ultrasound of the aorta, and femoral and popliteal arteries was conducted to measure flow velocities and occurrence of plaques.

### 2.4. Assessment of Additional Variables

The following variables were assessed at the baseline: age (in years), sex (dichotomized), education level (classified according to International Standard Classification of Education 97 (ISCED-97) [23]), body-mass-index (BMI in kg/m^2^), and history of ever smoking. Cardiovascular risk factors were as follows: diabetes (positive self-disclosure, taking medication of the A10 group (Anatomical Therapeutic Chemical Classification System (ATC-Code)), fasting glucose > 126 mg/dL, not fasting glucose > 200 mg/dL), coronary artery disease (CAD), and hypertension. Variables for PAOD included: ABI (as mean), carotid ultrasound and intima medial thickness, carotid artery plaque (yes/no). Blood samples were obtained for biomarker analysis (high-sensitive C-reactive protein (hs-CRP) and Interleukin 6 (IL-6)) and stored at −80 °C at the HCHS Biobank. Subsequently, plasma samples were analyzed via established enzyme-linked immunosorbent assays (ELISA).

### 2.5. Statistical Analysis

In descriptive analyses, medians with interquartile ranges (IQR) are presented for continuous variables. Similarly, proportions and 95% confidence interval (CI) are presented for categorical variables. Tests of normality were conducted using the Kolmogorov-Smirnov test. Descriptive analyses were applied for all variables stratified by the grading of periodontitis and PAOD. Multivariate analyses using logistic regression models were used to test hypotheses. Models with adjustments for clinically relevant confounders were applied after discussion and interpretation of the descriptive analyses (higher age, male sex, diabetes, low education, hypertension, high-sensitive CRP, and current smoking). Multiple imputation of ABI values per predictive mean matching based on higher age, male sex, intima media thickness, Fontaine stage, congestive heart disease, hypertension, dyslipidemia, smoking, and diabetes was performed. A *p* value of <0.05 was considered statistically significant. Statistical analyses were performed using R software, version 4.0.3.

## 3. Results

### 3.1. Descriptive Analyses

From 10,000 participants in the cohort, a subset of 3271 participants with complete vascular and oral examination were included in the analyses (Table 1). When comparing the stratum with both PAOD and severe periodontitis (n = 309) with the overall HCHS cohort, affected participants were more frequently males (56.0% vs. 48.9%; *p* < 0.001), were older (67 vs. 63; *p* < 0.001), exhibited a higher BMI (27.5 vs. 26.1; *p* < 0.001), a lower education (5.2% vs. 3.4%; *p* < 0.001), and were more frequently either current or former smoker (76.3% vs. 64.2%; *p* < 0.001).

Furthermore, the prevalence of diabetes (17.0% vs. 8.6%; *p* < 0.001), coronary artery disease (7% vs. 5%; *p* = 0.001), hypertension (79% vs. 66%; *p* < 0.001), and the occurrence of carotid artery plaques (47% vs. 31%; *p* < 0.001) were higher in PAOD patients with severe periodontitis when compared with the overall cohort.

Median hs-CRP differed significantly between controls with neither PAOD nor periodontitis and participants presenting both conditions (0.10 vs. 0.17). The same difference was apparent for IL-6 (1.27 vs. 1.97).

### 3.2. Multiple Logistic Regression Model

Associations of moderate and severe periodontitis and additional variables with PAOD were determined by logistic regression analysis. After adjusting for higher age, male sex, diabetes, low education, current smoking, hypertension, and hs-CRP, severe periodontitis was associated with PAOD (OR: 1.265; 95% CI: 1.006–1.591; *p* = 0.045) (Table 2). Additionally, higher age, female sex, diabetes, low education, hypertension, current smoking, and hs-CRP were associated with PAOD.

## 4. Discussion

The current study included well-characterized phenotypical data comprising 3271 participants with complete vascular and oral examination from a cross-sectional epidemiological cohort. When compared to the overall cohort, participants who were diagnosed with both PAOD and severe periodontitis were older, more often males, obese, current smoker, diabetics, and more often had coronary artery disease and hypertension. Furthermore, they exhibited a lower education level. After adjusting the model accordingly, a robust and independent association between severe periodontitis and PAOD was observed.

Few previous studies addressed the complex interaction between periodontitis and PAOD in a cross-sectional study design, such as the National Health and Nutrition Examination Survey (NHANES) (n = 3585) study [16] and the Korean Genome and Epidemiology Study (KoGES) (n = 1343) [24]. The association between those two common illnesses was also confirmed in a longitudinal study design within both the Health Professionals Follow-up Study (HPFS) [25] and in the Normative Aging Study (NAS) [26]. Notably, the comparison of the results from previous publications among themselves and with the current study appears challenging since different periodontal classification protocols were used. In the current study, we used a grading of periodontitis in severity grades based on a classification that is commonly accepted as gold standard [27].

To date, the pathogenic mechanism driving the association between periodontitis and PAOD is not clarified beyond doubt. Four different mechanisms were previously discussed [28].

(1) Periodontal bacteria enter the bloodstream or spread over the lymphatic system and invade the arterial wall, causing initiation of arteriosclerosis [29].
(2) Inflammatory mediators (Interleukin-6 and tumor-necrosis factor α) reaches the liver through the blood stream and induce an increased production of CRP and fibrinogen. Resulting an acute-phase reaction with pro-inflammatory and pro-atherogenic effect [30].
(3) Specific components of the oral biofilm (e.g., *porphyromonas gingivalis*) promote autoimmunity, which accelerates endothelial dysfunction [31].
(4) Bacterial toxins such as lipopolysaccharides (LPS), produced by oral pathogenic bacteria have pro-atherogenic and pro-inflammatory effects [28].

Interestingly, a longitudinal cohort study recently recruited 1002 patients with cardiovascular disease to assess the impact of subgingival pathogenic colonization on different cardiovascular endpoints, including stroke and myocardial infarction as well as death [32]. The population in that study was of similar age (69 years), but included more males (74%) and diabetics (36%) when compared with the current cohort. That said, differences between hospitalized patients and cohorts from epidemiological studies appear increasingly relevant: hospitalized patients were older, had a higher degree of vascular stenosis, and more frequently had diabetes and hypertension [33]. In contrast, the sample of our study was derived from a rather healthy population, even though the selected subset presented various cardiovascular comorbidities.

The current study has several strengths but also limitations. Although more than 6000 different information were collected from the participants included in the HCHS, a possible impact of non-observed confounding affecting the relationship between PAOD and periodontitis cannot be ruled out. In the current study, the model included both statistically and clinically relevant variables. The interesting fact that female sex was significantly associated with PAOD deserves a critical reflection. In contrast, data from previous studies suggested an association between male sex and PAOD. Beyond a possible modifying interaction between all variables in the current model, it appears likewise possible that the enrolled cohort differed from previous studies using hospitalized patients or cohorts under risk. Peculiarities of the study population in regard of health behavior and use of health services will be addressed in future studies.

Since periodontitis and PAOD are common illnesses with a long asymptomatic period, early intervention with preventive approaches is an emphasizing target. Patients with previous illnesses benefit from early diagnosis and consistent therapy, as chronic inflammation likely increases the risk of cardiovascular death [34]. According to valid practice guidelines [35], all patients with PAOD should be provided with an evidence-based best medical treatment including optimal pharmacological treatment and risk modification. Besides nutritional habits, smoking, and body weight, the oral hygiene and treatment of periodontitis may be an effective target to improve outcomes. The growing data on a possible association between periodontitis and PAOD on the one hand, and the comparatively minor invasiveness of a surgical treatment for periodontitis on the other hand give point to include dentists in the complementary treatment of vascular patients. Vice versa, an assessment of cardiovascular risk factors appears reasonable in patients treated for periodontitis. However, high-level evidence from appropriately powered comparative effectiveness trials is necessary to prove the safety and effectiveness beyond doubt.

## 5. Conclusions

In this cross-sectional analysis of a large cohort study, an independent association between periodontitis and PAOD was revealed. The results of the current study emphasize a potential for preventive medicine in an extremely sensitive target population. Future studies should determine the underlying factors modifying the relationship between both diseases.

## Figures and Tables

**Table 1:** Baseline characteristics from all (N = 10,000) participants and participants with complete vascular and oral examination (N = 3271) enrolled in the Hamburg City Health Study.

	Full Cohort	PAOD = No			PAOD = Yes		
	Overall	None/mild PD	Moderate PD	Severe PD	None/mild PD	Moderate PD	Severe PD
Number	10,000	437	1118	356	265	786	309
Male sex *	48.92 (47.94,49.9)	42.79 (38.24,47.47)	55.37 (52.44,58.26)	66.85 (61.81,71.54)	36.6 (31.03,42.56)	42.62 (39.21,46.11)	55.99 (50.41,61.41)
Age-y ^†^	63.00 (55.00,70.00)	57.00 (52.00,65.00)	61.00 (54.00,68.00)	64.00 (59.00,70.00)	60.00 (53.00,68.00)	63.00 (57.00,70.00)	67.00 (60.00,73.00)
BMI (kg/m^2^) ^†^	26.13 (23.53,29.21)	25.05 (22.87,27.87)	25.40 (23.33,28.57)	25.84 (23.89,28.42)	26.57 (23.54,29.61)	27.19 (24.52, 30.41)	27.50 (24.48, 30.47)
Low education level *	3.41 (2.33,4.5)	2.38 (0,7.53)	2.29 (0,5.47)	2.89 (0,8.53)	3.63 (0,10.17)	5.62 (2.05,9.4)	5.21 (0,11.3)
Medium education level *	52.38 (51.3,53.47)	44.89 (40.14,50.05)	44.31 (41.28,47.49)	55.2 (50,60.84)	58.06 (52.02,64.61)	60 (56.44,63.79)	59.03 (53.47,65.12)
High education level *	44.21 (43.13,45.3)	52.73 (47.98,57.89)	53.39 (50.37,56.57)	41.91 (36.71,47.54)	38.31 (32.26,44.85)	34.38 (30.82,38.17)	35.76 (30.21,41.85)
Ever smoking *	64.17 (63.22,65.1)	61.24 (56.59,65.69)	62.81 (59.94,65.6)	71.1 (66.17,75.59)	64.39 (58.45,69.93)	62.44 (58.98,65.77)	76.3 (71.24,80.71)
Diabetes *	8.62 (8.07,9.21)	5.98 (4.08,8.68)	4.22 (3.16,5.62)	5.12 (3.22,8.05)	8.4 (5.56,12.5)	11.64 (9.52,14.15)	17.06 (13.19,21.79)
CAD *	5.06 (4.64,5.51)	3.46 (2.11,5.64)	2.61 (1.83,3.73)	4.24 (2.58,6.87)	3.88 (2.12,6.99)	6.09 (4.61,8)	7.28 (4.86,10.78)
Hypertension *	66.12 (65.17,67.07)	51.06 (46.31,55.8)	60.11 (57.16,62.99)	67.15 (62.02,71.9)	59.3 (53.21,65.12)	71.32 (68.02,74.41)	79.33 (74.39,83.53)
High-sensitive CRP ^†^	0.12 (0.06,0.26)	0.10 (0.05,0.22)	0.11 (0.06,0.21)	0.11 (0.06,0.25)	0.12 (0.07,0.26)	0.15 (0.07,0.34)	0.17 (0.08,0.34)
IL-6 ^†^	1.64 (1.18,2.39)	1.27 (0.96,1.92)	1.42 (1.06,2.00)	1.59 (1.18,2.07)	1.63 (1.06,2.34)	1.71 (1.23,2.51)	1.97 (1.40,3.08)
IMT ^†^	0.75 (0.67,0.85)	0.72 (0.65,0.79)	0.74 (0.67,0.83)	0.78 (0.69,0.86)	0.74 (0.67,0.82)	0.77 (0.70,0.86)	0.81 (0.72,0.92)
Carotid plaque *	30.86 (29.94,31.79)	23.82 (20.01,28.1)	27.25 (24.68,29.98)	40.7 (35.64,45.96)	29.63 (24.24,35.65)	34.9 (31.56,38.39)	46.55 (40.89,52.3)
ABI, mean ^†^	1.03 (0.95,1.11)	1.07 (1.01,1.14)	1.06 (1.00,1.14)	1.05 (1.00,1.12)	0.93 (0.86,1.06)	0.92 (0.86,0.99)	0.89 (0.82,0.97)
DMFT-Index ^†^	20.00 (16.00,23.00)	17.00 (13.00,21.00)	19.00 (15.00,22.00)	20.00 (17.00,23.25)	18.00 (15.00,23.00)	20.00 (17.00,23.00)	22.00 (18.00,26.00)
BOP ^†^	7.69 (1.92,20.37)	3.57 (0.00,7.69)	7.69 (2.08,16.67)	19.25 (8.25,37.50)	1.92 (0.00, 6.60)	9.09 (3.33,19.23)	20.42 (10.00,39.17)
Oral Plaqueindex ^†^	8.70 (0.00, 29.17)	1.79 (0.00,12.85)	7.41 (0.00,24.00)	17.86 (4.17,46.67)	1.92 (0.00,14.79)	10.87 (0.00,31.82)	26.09 (7.41,64.44)
CAL ≥ 3 mm ^†^	36.54 (19.75,57.41)	13.10 (7.33,20.83)	36.31 (25.00,50.70)	67.96 (51.98,81.97)	12.92 (5.51,22.22)	40.97 (26.92,56.28)	71.88 (55.83,88.10)
Occurrence of both conditions		No PD, No PAOD					Both PD and PAOD

Results are presented as % with 95% confidence interval (*) or median with interquartile range (^†^) unless stated otherwise. Left column describes full cohort (N = 10,000). Analysis for association only included column PAOD = No and PAOD = Yes. PAOD = Peripheral arterial occlusive disease, PD = Periodontitis, BMI = Body-Mass-Index, CAD = Coronary Artery Disease, CRP = C-Reactive Protein, IL-6 = Interleukin-6, IMT = Intima Media Thickness, ABI = Ankle-Brachial-Index, DMFT-Index = Decayed, Missing, Filled Teeth- Index, BOP = Bleeding on Probing, CAL = Clinical Attachment Loss.

**Table 2:** Logistic regression model of variables significantly associated with a peripheral arterial occlusive disease in participants included in the Hamburg City Health Study.

Variable in the Model	Odds Ratio	2.5% CI	97.5% CI	*p* Value
Severe Periodontitis	1.265	1.006	1.591	0.045
Moderate Periodontitis	1.112	0.920	1.344	0.269
Higher Age (Increase per Year)	1.021	1.009	1.033	0.001
Diabetes Mellitus	1.859	1.439	2.401	<0.001
Low Education	1.639	1.084	2.479	0.020
Hypertension	1.432	1.2110	1.693	<0.001
High-sensitive CRP	1.277	1.069	1.524	0.007
Current Smoking	1.217	1.007	1.472	0.042
Male Sex (vs. female sex)	0.628	0.533	0.741	<0.001

CI = Confidence Interval, CRP = C-Reactive Protein.

## Data Availability

The data presented in this study are available on request from the corresponding author. The data are not publicly available due to legal restrictions.
